# Analysis of m^6^A modulator-mediated methylation modification patterns and the tumor microenvironment in lung adenocarcinoma

**DOI:** 10.1038/s41598-022-20730-6

**Published:** 2022-11-30

**Authors:** Qing-Cui Zeng, Qin Sun, Wen-Jie Su, Jia-Cen Li, Yi-Sha Liu, Kun Zhang, Li-Qing Yang

**Affiliations:** 1 Department of Geriatric Intensive Care Unit, Sichuan Academy of Medical Sciences and Sichuan Provincial People’s Hospital, Chengdu, China; 2 Department of Anesthesiology, Sichuan Academy of Medical Sciences and Sichuan Provincial People’s Hospital, Chengdu, China; 3 Department of Pathology, Sichuan Academy of Medical Sciences and Sichuan Provincial People’s Hospital, Chengdu, China; 4 Department of Chest Surgery, Sichuan Academy of Medical Sciences and Sichuan Provincial People’s Hospital, Chengdu, China; 5 Department of Respiratory Medicine, Eastern Hospital, Sichuan Academy of Medical Sciences, Sichuan Provincial People’s Hospital, Sichuan Province, No. 585, Honghe North Road, LongQuanYi District, Chengdu, 610000 China; 6 Chinese Academy of Sciences Sichuan Translational Medicine Research Hospital, Chengdu, China

**Keywords:** Cancer, Computational biology and bioinformatics

## Abstract

Lung adenocarcinoma (LUAD) is the most common histological subtype of lung cancer. In the development and progression of LUAD, epigenetic aberration plays a crucial role. However, the function of RNA N6-methyladenosine (m^6^A) modifications in the LUAD progression is unknown. The m6A regulator modification patterns in 955 LUAD samples were analyzed comprehensively. Patterns were systematically correlated with the tumor microenvironment (TME) cell-infiltration characteristics. Using principal component analysis algorithms, the m6Ascore was generated to quantify m^6^A modification patterns in individual tumors. Then, their values for predicting prognoses and therapeutic response in LUAD patients were assessed. Three distinct m^6^A modification patterns in LUAD were identified. Among them, the prognosis of m6Acluster C was the best, while the prognosis of m^6^Acluster A was the worst. Interestingly, the characterization of TME cell infiltration and biological behavior differed among the three patterns. To evaluate m^6^A modification patterns within individual tumors, an m^6^Ascore signature was constructed. The results showed that the high m^6^Ascore group was associated with a better prognosis; tumor somatic mutations and tumor microenvironment differed significantly between the high- and low- m^6^Ascore groups. Furthermore, in the cohort with anti-CTLA-4 treatment alone, patients with a high m^6^Ascore had higher ICI scores, which indicated significant therapeutic advantage and clinical benefits.

Globally, cancer-related morbidity and mortality rate are increasing rapidly. Among them, lung cancer is the leading cause of cancer-related mortality^1–3^. Non-small cell lung cancer (NSCLC) is the most common type of lung cancer, accounting for about 85% of all cases^4–6^. Lung adenocarcinoma (LUAD) is one of the major subtype of NSCLC, accounting for roughly 40% of all lung cancer cases^7,8^. In recent years, surgical resection, targeted therapy, chemotherapy, and other therapeutic approaches have been proven to improve the survival of NSCLC patients, but the prognosis remains poor with many limitations^9–11^. Therefore, further research is required to understand the underlying tumor biological process and treatment options for LUAD.

N6-methyladenosine(m^6^A)RNA methylation is regarded to be one of the most significant and abundant forms of RNA modification in eukaryotic cells, which plays a key regulatory role in the cell’s existence^12–14^. The enzymes with writers (methylases), erasers (demethylases), and readers are mostly involved in m^6^A modifications, representing 0.1%-0.4% of the total adenosine residues. Numerous pieces of researchers have revealed that m^6^A regulatory factors play important roles in several cancer-related biological processes, including apoptosis, cell proliferation, invasion, and metastasis^15–17^. Zhang et al. demonstrated that the reduction of m^6^A methylation of RNA activates the oncogenic Wnt/PI3K-Akt signaling pathway, which can promote the malignant phenotype of gastric cancer cells^18^. By regulating the m^6^A level of USP7 mRNA, the m^6^A demethylase FTO can promote lung cancer cell proliferation^19^. According to Xu et al., the m^6^A methyltransferase METTL3 promotes cell proliferation by inhibiting SOCS2 to maintain the tumorigenicity of colon cancer. However, the function of m^6^A modulators in LUAD is still unclear^20^. Thus, in-depth studies and further investigations are required to understand the underlying mechanism for the m^6^A regulators in LUAD.

Bioinformatics analysis based on database mining is regarded to be one of the most promising approaches for cancer translational research, with the advancement of gene sequencing technology and the establishment of tumor databases^21–23^. In the current investigation, the leading goal was to evaluate the correlation between the m^6^A modification pattern and the tumor microenvironment. We revealed three different m^6^A modification patterns and assessed the clinical features, prognostic value, and immune infiltration of the resulting m^6^A clusters. In addition, we established a scoring system to quantify the m^6^A modification patterns and determine its value in predicting the prognosis and therapeutic response of LUAD patients.

## Materials and methods

### LUAD data source and preprocessing

The Cancer Genome Atlas (TCGA) and Gene-Expression Omnibus (GEO) database were used to acquire gene expression data and clinical annotations for LUAD samples. This analysis was comprised a total of 955 LUAD cases (TCGA-LUAD: 513, GSE68465: 442) sourced from TCGA and GEO databases. Patients without survival information were excluded from this study. In TCGA-LUAD cohorts, fragments per kilobase million (FPKM) were transformed into transcripts per million (TPM) values^24,25^. Before model validations, normalized expression values were logarithmically transformed and scaled for GEO data sets. The "sva" package in R software was used to examine the batch effect^26,27^. The clinical information of patients is provided in Table S1.

### Unsupervised clustering for m^6^A regulators

The m^6^A -related literature revealed twenty-three m^6^A regulators, including 8 writers (METTL3, METTL14, METTL16, WTAP, VIRMA, ZC3H13, RBM15, RBM15B), 13 readers (YTHDC1, YTHDC2, YTHDF1, YTHDF2, YTHDF3, HNRNPC, FMR1, LRPPRC, HNRNPA2B1, IGFBP1, IGFBP2, IGFBP3, RBMX), and 2 erasers (FTO, ALKBH5)^28–30^. Univariate Cox model was used to analyze the correlation between m^6^A regulatory variables and prognosis. To identify the different m^6^A modification patterns and classify patients, the "ConsensuClusterPlus" package was used to conduct the above steps and 1000 times repetitions for guaranteeing the stability of clustering^31^.

### Gene set variation analysis (GSVA) and functional annotation

To study the differences in biological processes between m^6^Aclusters and m^6^A modification patterns, the "GSVA" R package was used to perform GSVA enrichment analysis^32–34^. The R package "clusterProfiler" was used for functional annotation and the gene set file (c2.cp.kegg.v7.2.symbols.gmt) was obtained from the MSigDB database (https://www.gsea-msigdb.org), with the cutoff value of FDR < 0.05.a.

### Immune cell infiltration estimation

To assess the relative abundance of each cell infiltration in distinct m6A subtypes and the amount of immune cell infiltration we employed the ssGSEA (single-sample gene-set enrichment analysis) program^35,36^.

### Gene ontology (GO) and Kyoto Encyclopedia of Genes and Genomes (KEGG) pathway enrichment analyses

Using the "clusterProfiler" (version 3.0.4; https://www.rdocumentation.org/packages/clusterProfiler/versions/3.0.4), "enrichplot" (version 1.13.1.994; https://www.rdocumentation.org/packages/enrichplot/versions/1.13.1.994) and "ggplot2" (version 3.3.5; https://www.rdocumentation.org/packages/ggplot2/versions/3.3.5) packages of R software, GO and KEGG enrichment analysis on the gene set was performed^37,38,49–52^.

### Generation of m6Ascores

We constructed a set of scoring system to evaluate the m^6^A modification pattern of individual patients with LUAD-the m^6^A gene signature, and we termed it as m^6^Ascore. The process of establishing the m^6^A scoring system was as follows: the DEGs identified from different m^6^Aclusters were firstly normalized among all samples and the overlap genes were extracted. Differential analysis and Venn diagram showed that there are 15 common differential genes among the three m^6^Aclusters. Then, we performed univariate Cox regression analysis for each gene. These genes with a significant prognosis were extracted for the next step of the analysis. Then we perform principal component analysis (PCA) to calculate the m^6^Ascore using the following formula:

$${\text{m}}^{{6}} {\text{Ascore }} = \, \sum \left( {{\text{PC1}}_{{\text{i}}} + {\text{ PC2}}_{{\text{i}}} } \right)$$

where i is the expression of the m^6^A phenotype-associated genes^39,40^.

### Generation of ImmuneScore, StromalScore, and ESTIMATEScore

Through the "estimate" package of R software the ratio of the immune stromal components of each sample in the tumor microenvironment was estimated, and ImmuneScore, StromalScore, and ESTIMATEScore, which was positively correlated with the ratio of immune, stromal, and the sum of both, respectively^41,42^.

### Correlation of m^6^A-scoring signature with genome mutations, clinical information, and Immunity

The associations between the high- and low-m^6^Ascore groups and mutation and clinical status were investigated based on the m^6^A-scoring signature. In addition, ssGSEA was used to quantify the subset of tumor-infiltrating immune cells between the two groups and to assess their immunological differences. The Cancer Immunome Database (TCIA) was used to download the Immune checkpoint inhibitor (ICI) Immunophenoscore (IPS) for immunotherapy. IPS is a good predictor of CTLA-4 and PD-1 blocking responsiveness and thus was used to predicts the response to immunotherapy between the two groups^43,44^.

### Statistical analysis

A t-test was used for variables with a normal distribution, and a non-parametric test (Wilcoxon rank rank-sum test) was employed for variables with a non-normal distribution when comparing data between two groups. One-way ANOVA and Kruskal–Wallis tests were used as parametric and non-parametric methods, respectively for data from more than two groups. A Chi-square test was used to examine the correlation between m^6^A modification patterns and clinical features. The *P* values were corrected for multiple comparisons via the Benjamini and Hochberg (BH). The best cut-off score between the two groups of high and low m^6^Ascore was derived by the surv-cutpoint function. The mutation landscape in patients was shown using the waterfall function of the "maftools" package. The R packages "survival" and "survminer" were used for survival analysis^45^. Unless specified, *P*-value < 0.05 was statistically significant. All data processing was done in R 4.1.0 software.

## Results

### The landscape of genetic variation of m^6^A regulators in LUAD

**Figure 1:**
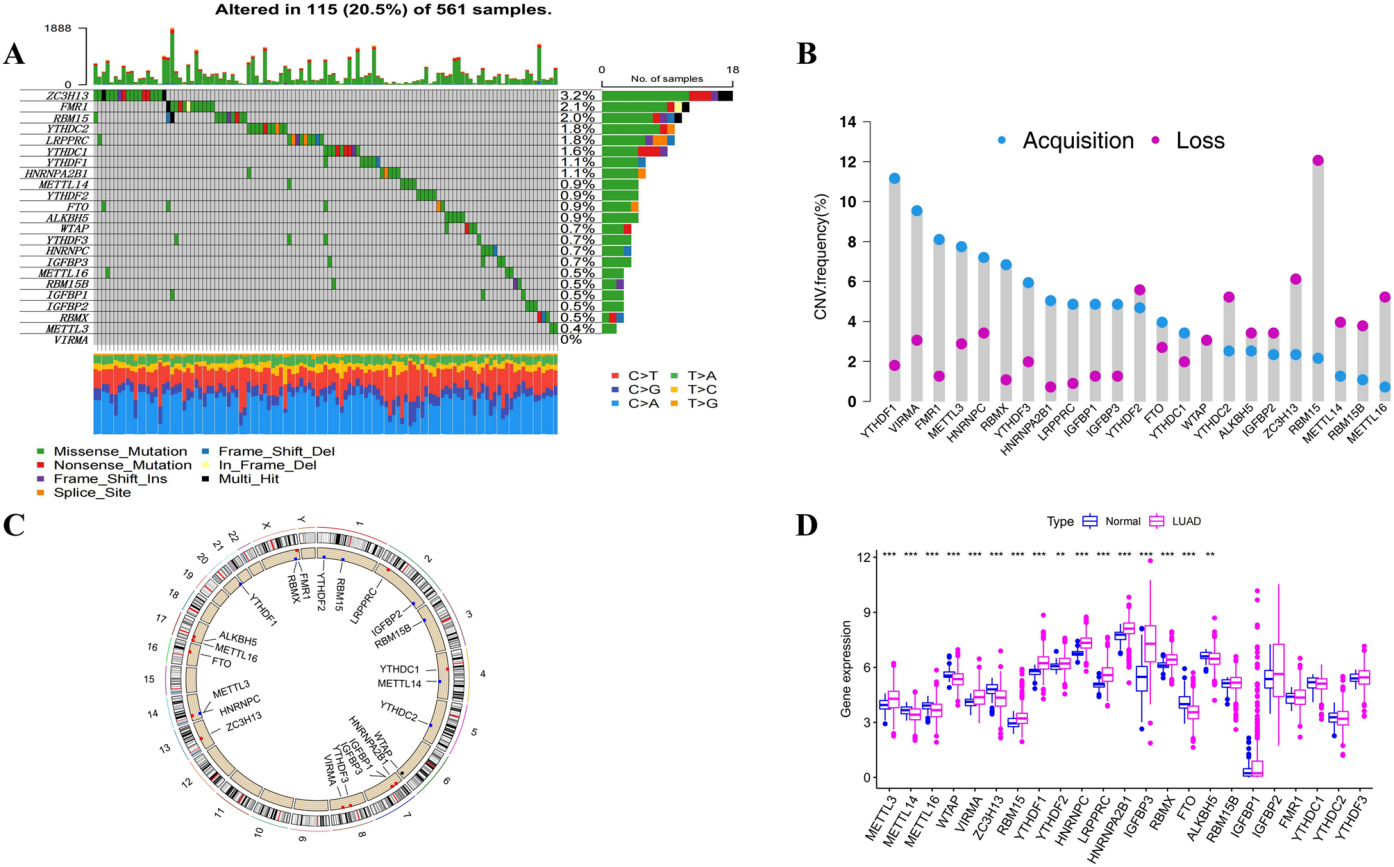
Genetic variation of m^6^A regulators in LUAD. (**A**) Genetic alteration for the queried m^6^A regulators. (**B**) Frequency of CNVs in m^6^A regulators. Blue dots represent CNV amplification; purple dots represent CNV deletion. (**C**) The location of the CNV alteration of the m^6^A regulators changes on 23 chromosomes in the TCGA-LUAD cohort. Red square represent more samples with increased copy number of the gene. Blue square represent more samples with missing copy number of the gene. (**D**) Comparison of gene expression of 23 m^6^A regulators in LUAD with normal tissue (*P < 0.05; **P < 0.01; ***P < 0.001). LUAD : Lung adenocarcinoma.

The flow chart diagram of this study is presented in Fig. S1. The somatic mutations and copy counts of 23 m^6^A regulators were summarized in LUAD. Among 561 samples, we found 115 experienced mutations of m^6^A regulators, with a frequency of 20.5%. The mutation frequency of ZC3H13 was highest, followed by FMR1 (Fig. 1A). Further analysis showed that there was no significant mutation co-occurrence relationship between ZC3H13 and other m^6^A regulators (Fig. S2). The study of CNV alteration frequency revealed that 23 regulators had a common CNV modification, with the majority of them focusing on copy number amplification, while RBM15, ZC3H13, METTL16, and YTHDC2 had a high frequency of CNV deletion (Fig. 1B). The location of CNV alterations on the chromosome for m^6^A regulators is shown in Fig. 1C. In addition, compared with normal tissues, the expression levels of METL3, VIRMA, RBM15, YTHDF1, YTHDF2, LRPPRC, HNRNPA2B1, IGFBP3, RBMX, FTO, and ALKBH5 were significantly up-regulated in LUAD, and the median value of the boxplot was higher, WTAP, METTL16, METTL14, and ZC3H13 were significantly down-regulated (*P* < 0.05, Fig. 1D).

### m^6^A RNA methylation modification patterns mediated by 23 regulators in LUAD

**Figure 2:**
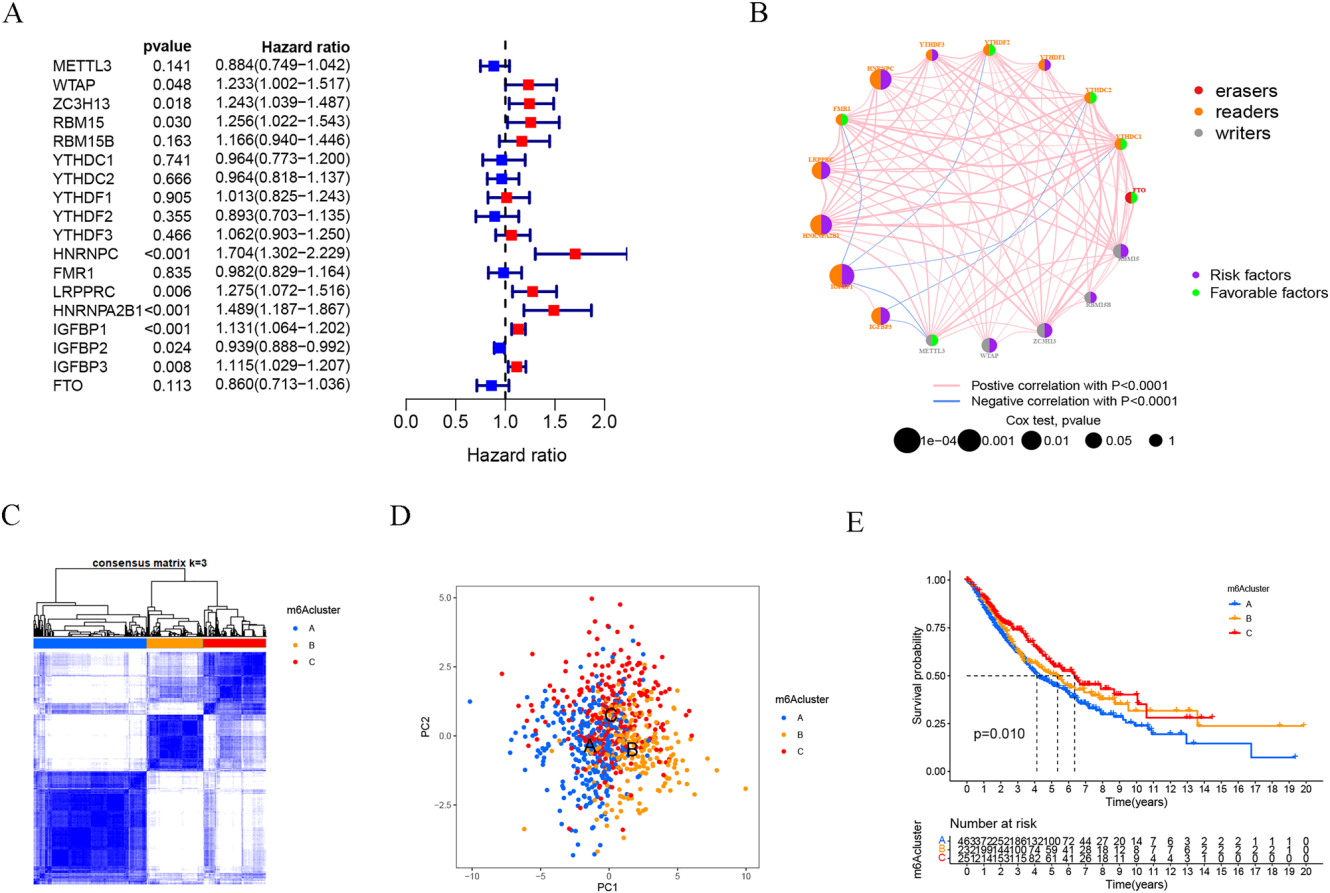
m^6^A RNA methylation modification patterns mediated by 23 regulators in LUAD. (**A**) Univariate Cox regression models were used to assess the prognoses based on 23 m6A regulators. (**B**) Interactions between m6A regulators in LUAD. The size of the circles represents the effect of each modulator on prognosis; larger circles represent a greater effect on prognosis (p-values: 1–0.0001). The association between the m6A regulators is shown by the connecting line; negative correlations are in blue and positive correlations are in pink. (**C**) Heat map of the matrix of co‐occurrent proportions for LUAD samples (K = 3). (**D**) Principal component analysis (PCA) analysis of m6A methylation modification pattern. (**E**) The overall survival of m6A methylation modification pattern using Kaplan–Meier curves.

One meta-cohort was formed by combining two LUAD datasets (TCGA-LUAD, GSE68465) with existing OS data and clinical information (Table S1). The univariate Cox regression analysis was used to screen for m^6^A regulators associated with prognosis in LUAD (Fig. 2A). The findings revealed that WTAP, ZC3H13, RBM15, HNRNPC, LRPPRC, HNRNPA2B1, IGFBP1, and IGFBP3 were risk factors for poorer prognosis. The interaction between the m^6^A regulators is displayed in Fig. 2B. Based on the expression levels of 23 m6A regulatory genes, the "ConsensusClusterPlus" R package was used to classify patients with qualitatively different m6A modification patterns, K = 3 is the optimal number of clusters determined by the consensus clustering algorithm, and three different modification patterns were determined (Fig. 2C), named m^6^Acluster A, m^6^Acluster B, and m^6^Acluster C. Furthermore, it was also found that most of the three m^6^A modified subtypes are in a state of separation, but there is also some overlap in the middle. Therefore, the PCA method based on m^6^A-related genes may have limitations for some patients, which requires our attention (Fig. 2D). In the results of prognostic analysis, we found that m^6^Acluster C has a higher 50% survival rate and a better survival advantage (*P* = 0.01, Fig. 2E).

### TME cell infiltration characteristics in distinct m^6^A modification patterns

**Figure 3:**
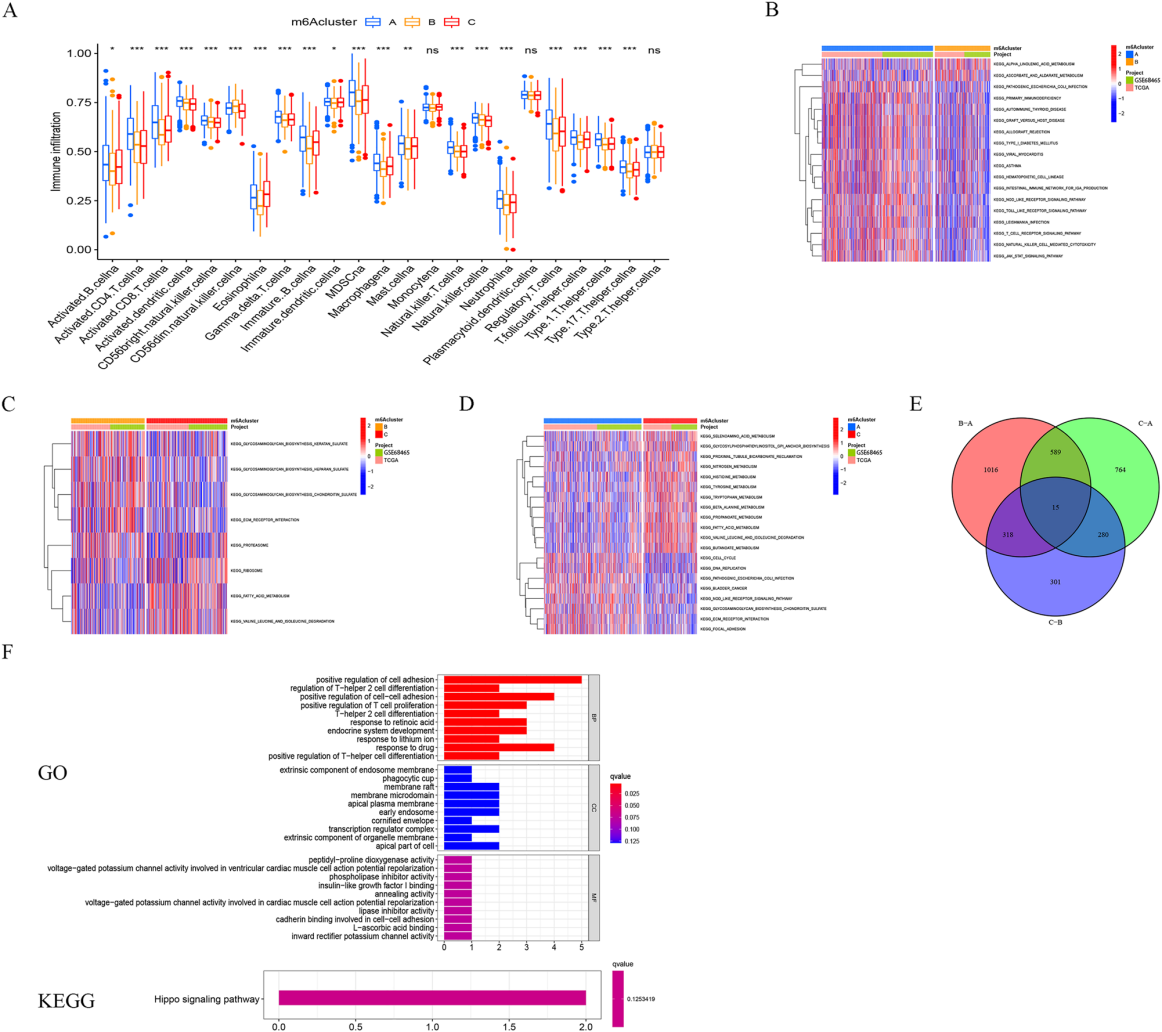
TME cell infiltration characteristics in distinct m^6^A modification patterns. (**A**) The abundance of TME infiltrating cell types in three m^6^A modification patterns. Statistical p-values are indicated by asterisks (*P < 0.05; **P < 0.01; ***P < 0.001). (**B**–**D**) GSVA enrichment analysis showing the activation states of biological pathways in different m^6^A modification patterns. Heat map for the biological processes; red represents activated pathways and blue represents inhibited pathways. (**E**) Heat map showing overlapping genes for three m^6^A methylation modification pattern subtypes. (**F**) Results of GO (up) and KEGG (down) enrichment.

To further analyze the difference in immune cell infiltration between different m^6^A modification patterns, the ssGSEA algorithm was used. It was found that the three m^6^A modification modes have significant differences in the degree of enrichment of immune cell infiltration. m^6^Acluster A has more abundant immune cell infiltration, with the highest median boxplot, while m6Acluster B has the worst level (Fig. 3A). Among the three m^6^A modification modes, m^6^Acluster A had a higher abundance of immune infiltrating cells, including Activated B cell, Activated CD4 T cell, Activated CD8 T cell, Activated dendritic cell, CD56bright natural killer cell, Immature dendritic cell, MDSC, Macrophage, Neutrophil, Type 1 T helper cell, and Type 17 T helper cell (Fig. 3A). Patients with this m^6^A modification pattern (m^6^Acluster A) had the poorest prognosis compared to the other two subtypes of m^6^A modification patterns (Fig. 2E). Meanwhile, GSVA enrichment analysis was performed to explore the biological behaviors between the m^6^A modification patterns (Fig. 3B-D). We found that immune-related pathways such as the T cell receptor signaling pathway, toll-like receptor signaling pathway, and natural killer cell-mediated cytotoxicity are significantly enriched in m^6^Acluster A (Fig. 3B). Glycosaminoglycan biosynthesis-related pathways and ECM receptor interaction were all substantially abundant in m^6^Acluster B (Fig. 3C). m^6^Acluster C was significantly enriched in pathways related to metabolism (Fig. 3D).

In addition, 15 overlapping m^6^A phenotype-related DEGs (IGFBP2, BIRC3, ASCL1, RET, IL18, KCNH2, PIGT, ANXA1, MYOF, RPRM, TMEM59L, SOX2, EGLN3, MYO5C, and FCN3) in the three m^6^A modification patterns subtypes were identified in this study and performed GO and KEGG enrichment analysis (Fig. 3E). Positive regulation of T − helper cell differentiation, T − helper 2 cell differentiation, regulation of T − helper 2 cell differentiation, positive regulation of T cell proliferation, positive regulation of cell − cell adhesion, positive regulation of cell adhesion, transcription regulator complex, cadherin binding involved in cell − cell adhesion, and Hippo signaling pathway were all enriched in these DEGs (Fig. 3F).

### Generation of m^6^A gene signatures and m^6^Ascore

**Figure 4:**
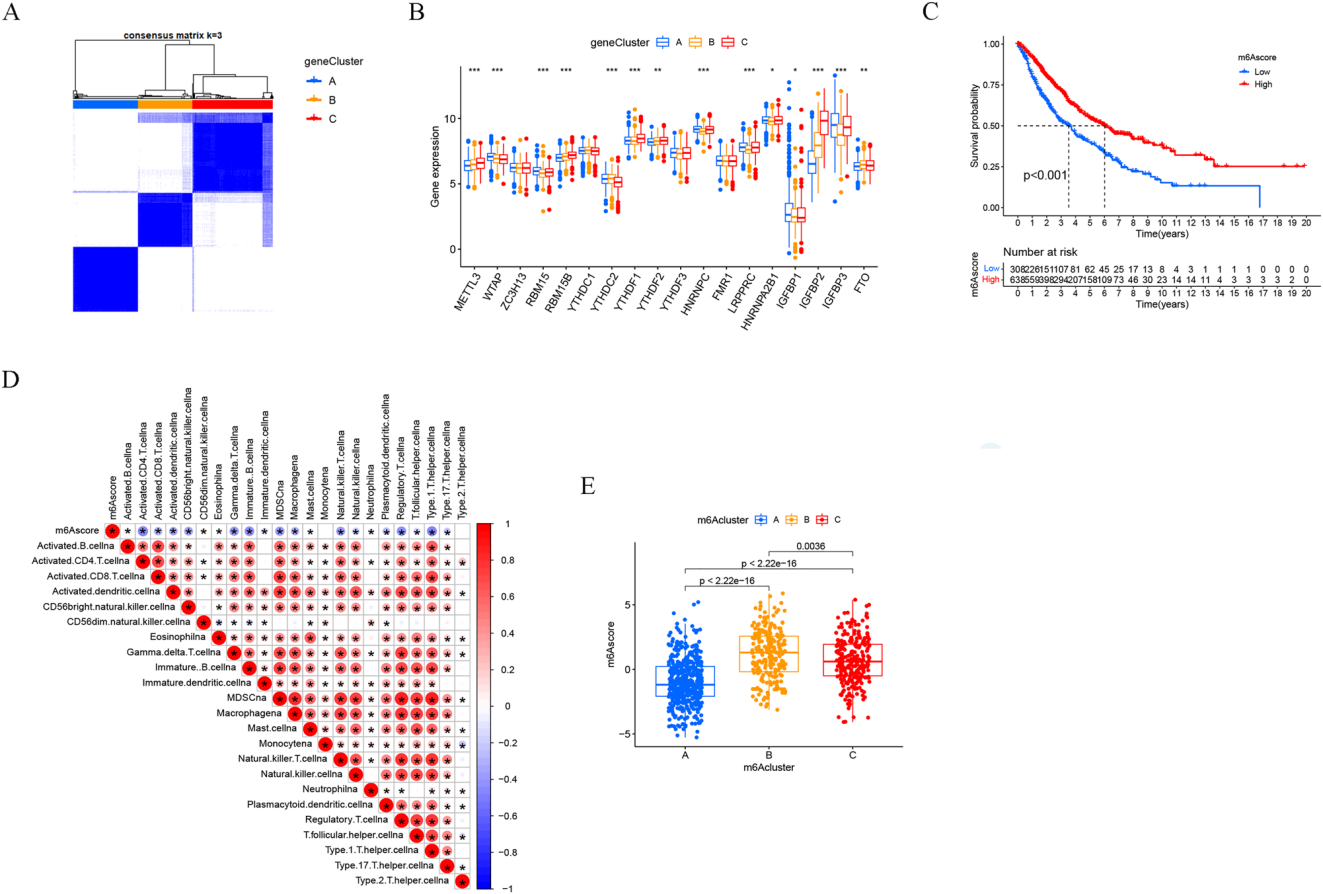
Generation of m^6^A signatures and m^6^Ascores. (**A**) Consensus clustering of genecluster for k = 3. (**B**) Gene expression levels of 23 m6A regulators in three m6A modification genomic phenotypes (*P < 0.05; **P < 0.01; ***P < 0.001). (**C**) The overall survival of m6A scoring signature. (**D**) Correlation between m6Ascore and immune cell infiltration. (**E**) m6Ascore in different m6Acluster subgroups.

Based on the DEGs between the three m^6^Aclusters, an unsupervised cluster analysis (Fig. 4A) was performed and three m^6^A modified genome phenotypes, named geneCluster A, geneCluster B, and geneCluster C. Substantial variations in the expression of m^6^A regulators across these three m^6^A-modified genomic phenotypes were found (Fig. 4B). geneCluster C had considerably greater levels of METTL3, RBM15B, YTHDF1, YTHDF2, YTHDF3, and IGFBP2 than the other two groups. In addition, a scoring system was devised to determine the pattern of m^6^A modification in each LUAD patient. To investigate the relationship between m^6^Ascore and patient prognosis, the "survminer" program was used to obtain the optimum cut-off value and classify patients into high- and low- m^6^Ascore groups. The m6Ascore group clinical information is shown in table S2. The 50% survival of the high m^6^Ascore group was significantly higher than that of the low m^6^Ascore group, and the high m^6^Ascore was associated with a better prognosis (*p* < 0.001, Fig. 4C). Most immune cells have a negative association with m^6^Ascore, according to the findings (Fig. 4D). According to the results of the boxplot, we can find that the median line value of the m^6^Acluster A group is the lowest, and the patients in the m^6^Acluster A group have a lower m6ascore and a poor prognosis (Fig. 4E).

### Clinical and tumor somatic mutation characteristics of m^6^Ascore cluster in TCGA-LUAD cohort

**Figure 5:**
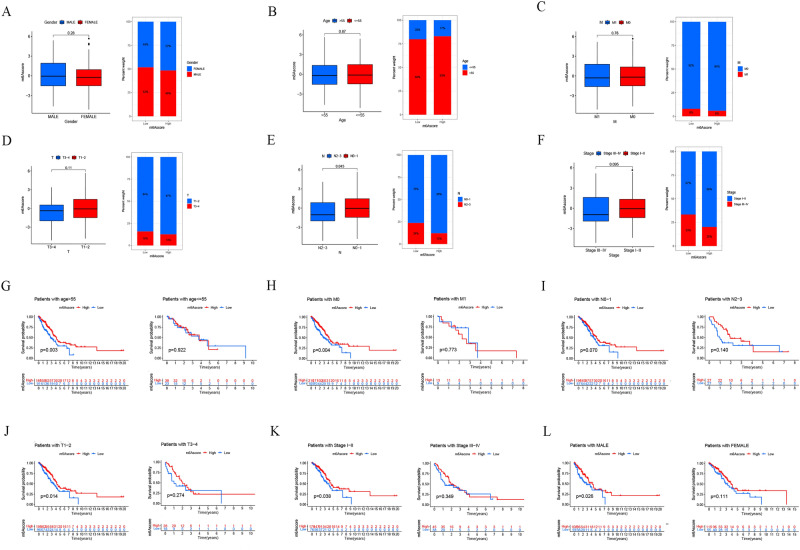
Clinical characteristics of m^6^Ascore cluster in TCGA-LUAD cohort. (**A**–**F**) Correlation analysis between clinical characteristics and m^6^Ascore. (**G**–**L**) Correlation analysis between m^6^Ascore and prognosis in different patient types.

To further analyze the clinical characteristics based on the m^6^Ascore, the clinical information of LUAD patients from the TCGA database was obtained (Table S1, S2). The results showed that m^6^Ascore were higher in the N0-1 stage (*p* = 0.045, Fig. 5E) and were not significantly correlated with the other clinical stages (Fig. 5A,B,C,D,F). In addition, the association between different types of patients and their prognoses was examined (Fig. 5G–L), and the results showed that a high m^6^Ascore was related to a better prognosis in most patient categories (age > 55, M0, T1-2, Stage1-2, MALE). Another important finding is that m^6^Ascore can be used as an independent prognostic indicator for LUAD patients (Fig. S3), and a higher m^6^Ascore is associated with a better prognosis, which is also consistent with our previous findings (Fig. 4C).

**Figure 6:**
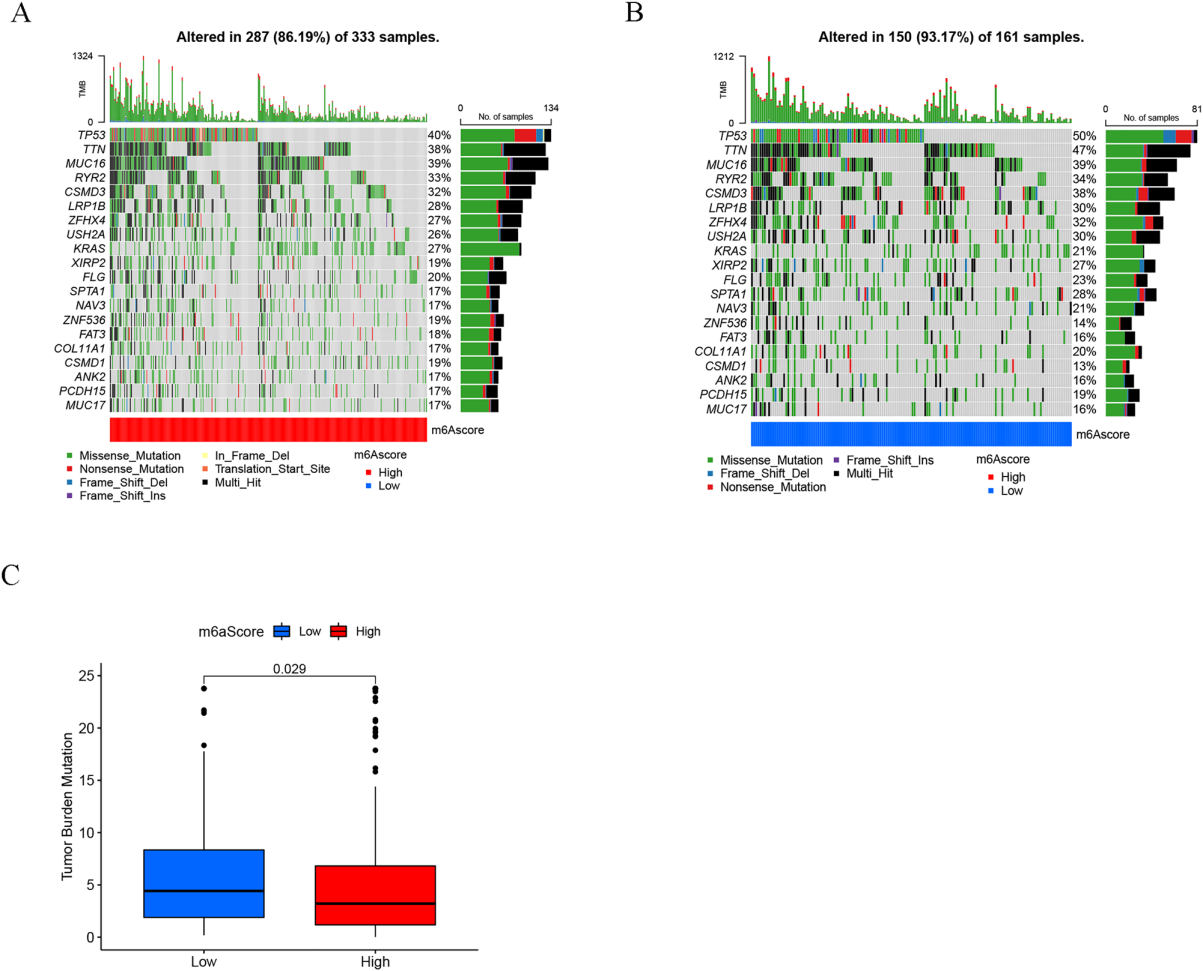
Characteristics of tumor somatic mutation in the m^6^A score cluster in the TCGA dataset. (**A**) Waterfall plot of tumor somatic mutation for high m6A score. (**B**) Waterfall plot of tumor somatic mutation for low low m6Ascore. (**C**) The tumor burden mutation level was higher in the low m6Ascore group.

Then, in the TCGA-LUAD cohort, the "maftools" program was utilized to evaluate the differences in somatic mutation distribution between low- (Fig. 6B) and high-m^6^Ascores (Fig. 6A). The results showed that the low m^6^Ascore group had more extensive tumor burden mutation than the high m^6^Ascore group (*p* = 0.029, Fig. 6C), and the somatic mutation rate of TP53 in the low m^6^Ascore group was higher.

### The role of m^6^Ascore in tumor microenvironment and immunotherapy

**Figure 7:**
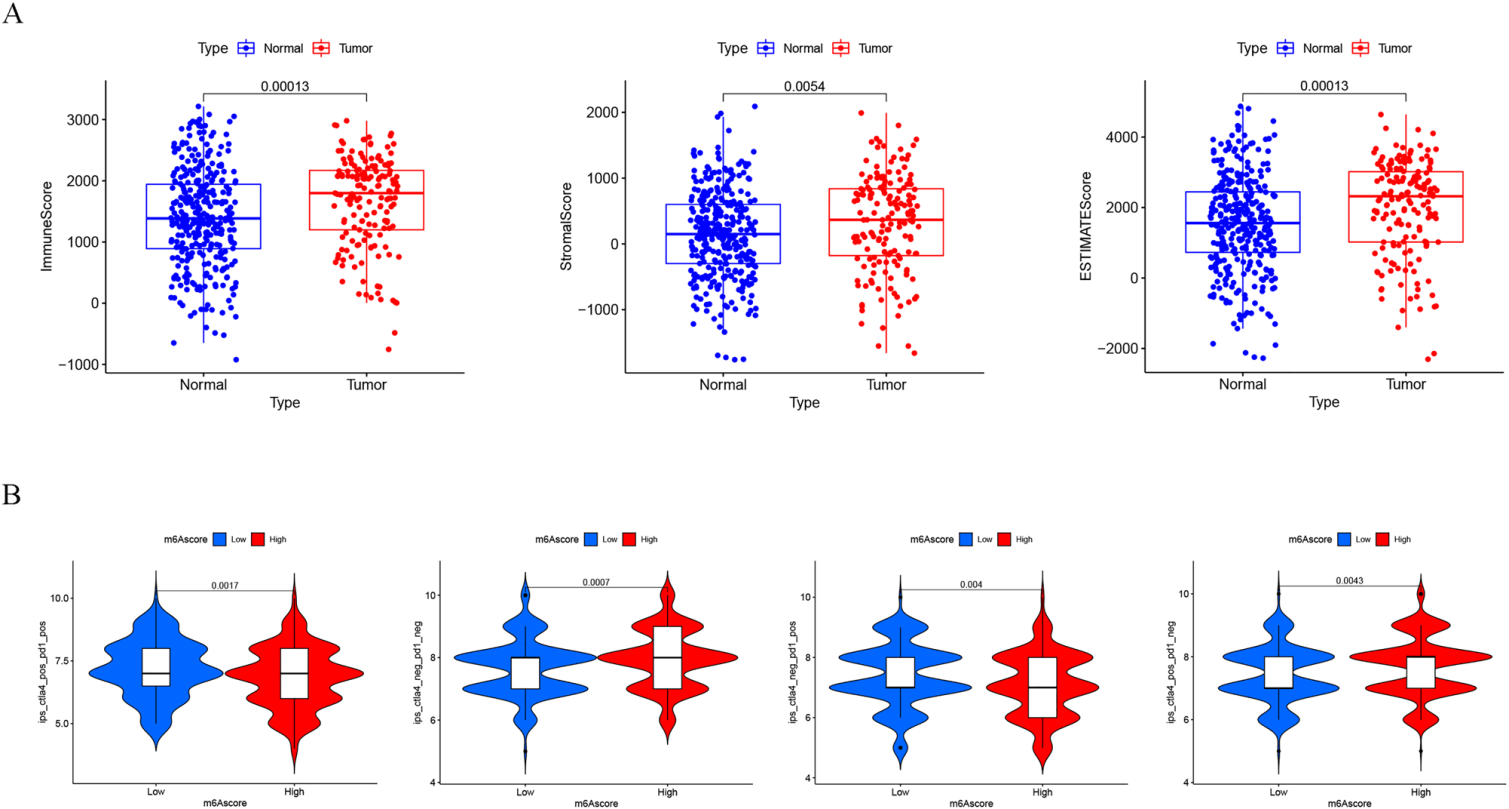
The role of m6Ascore in tumor microenvironment and immunotherapy. (**A**) The score levels of ImmuneScore, StromalScore, and ESTIMATEScore in the high- and low- m6Ascore groups. (**B**) The relative distribution of immunophenoscore (IPS) was compared between low- and high- m6Ascore groups.

To investigate the function of the m^6^Ascore in the tumor microenvironment, the R package "estimate" was used to calculate the proportion of immune matrix components in each sample in the tumor microenvironment. An important finding was that compared with the low m^6^Ascore group, the boxplot of the high m^6^Ascore group has a significantly higher midline, indicating that the high m^6^Ascore group has higher scores of ImmuneScore, StromalScore, and ESTIMATEScore (Fig. 7A). The results of clinical correlation analysis showed that with the occurrence and development of tumors (Stage I–IV, T1-T4, M0–M1), the ImmuneScore, StromalScore, and ESTIMATE Score decreased significantly (Fig. S4). In addition, the immunotherapy response of ICI treatment represented by the CTLA-4/PD-1 inhibitors in the high- and low-m^6^Ascore groups was investigated (Fig. 7B). The results showed that patients in the high m^6^Ascore group had higher ICI scores in the anti-CTLA-4 treatment alone cohort. In both the anti-PD-1 therapy alone and the combination of the anti-CTLA-4/PD-1 treatment cohorts, patients with a low m^6^Ascore had higher ICI scores.

## Discussion

m^6^A methylation is the most common form of mRNA modification which plays an important role in the development and progression of cancer by interacting with several m^6^A modulators. Previous studies showed that m^6^A modulator modification is significantly correlated with inflammation, tumor microenvironment, and immune response^46–48^. Thus, investigating the involvement of various m^6^A modification mechanisms in LUAD will further enhance the understanding of its occurrence and development. Methylation modification patterns, tumor microenvironment infiltration, and the characterization of m^6^A modulators in LUAD may help in determining the potential prognostic characteristics of cancer and aid in the development of novel therapeutic methods.

In the current study, 23 m^6^A regulatory factors from m6a-related literature were collected, and then their expression levels, mutations, and prognosis in LUAD were explored. It was observed that ZC3H13 (writers) showed the highest mutation frequency, followed by FMR1 (readers). Upon comparing with normal tissues it was found that METTL3, VIRMA, RBM15, YTHDF1, YTHDF2, LRPPRC, HNRNPA2B1, IGFBP3, RBMX, FTO, and ALKBH5 were significantly up-regulated in LUAD tissues, while WTAP, METL16, METL14, and ZC3H13 were significantly down-regulated. WTAP, ZC3H13, RBM15, HNRNPC, LRPPRC, HNRNPA2B1, IGFBP1, and IGFBP3 were considered to be the risk factors for poor prognosis. In addition, based on 23 m^6^A regulatory variables, three distinct m^6^A methylation modification patterns in LUAD were discovered. The best prognosis among these three models was m^6^Acluster C, whereas, the worst prognosis was m^6^Acluster A. Interestingly, these three models varied TME cell infiltration characteristics and biological behaviors. Activated B cell, Activated CD4 T cell, Activated CD8 T cell, activated dendritic cell, CD56 bright natural killer cell, immature dendritic cell, MDSC, Macrophage, Neutrophil, Type 1 T helper cell, and Type 17 T helper cell were found to be prevalent in innate immune cell infiltration. However, patients with this m^6^A modification pattern showed the worst prognosis compared to the other two patterns. The results of GSVA analysis revealed that m^6^Acluster A is associated with immune-related pathways, such as primary immunodeficiency, autoimmune thyroid disease, allograft rejection, nod-like receptor signaling pathway, toll-like receptor signaling pathway, t-cell receptor signaling pathway, natural killer cell-mediated cytotoxicity, and jak stat signaling pathway. This might explain the poor prognosis of m^6^Acluster A. Some comprehensive pathways were enriched by m^6^Acluster B, and the tumor metabolism-related pathways were highly represented by m^6^Acluster C. Based on the above-mentioned m^6^A modification patterns in LUAD, the m^6^A-related transcriptional expression patterns in these modifications were further explored and it was identified that 15 m^6^A phenotypic DEGs were present. These genes were significantly associated with immune-related biological pathways, according to the results of GO and KEGG enrichment analyses. Following that, three genomic subtypes based on m^6^A characteristic genes were identified in this study. These subtypes were also linked to the matrix and immunological activation, demonstrating the importance of m^6^A modification in creating distinct TME landscapes. TME plays an important role in the initiation and progression of tumorigenesis. The function of m^6^A regulatory factors in the immune microenvironment of LUAD to modify TME should be investigated further for the discovery of novel potential therapeutic targets.

Different patients had varying levels of m^6^A modifications. Thus, a scoring system (m^6^Ascore) was established to quantify the m^6^A modification pattern of patient tumors. It was observed that the high m^6^Ascore group was related to a better prognosis and reduced mutation frequency. According to the tumor microenvironment analyses the high m^6^Ascore group revealed higher ImmuneScore, StromalScore, and ESTIMATEScore. It was worth noting that the high expression levels of ImmuneScore, StromalScore, and ESTIMATEScore in LUAD patients were all associated with a better prognosis. The immunological checkpoints cytotoxic T lymphocyte-associated antigen 4 (CTLA-4) and programmed death 1 (PD-1) have provided novel tools for immunotherapy. Immune checkpoint inhibitors (ICI) acted against these molecules by relieving inhibition of certain pathways, thereby strengthening the immune system to produce anti-tumor effects. Therefore, the efficacy of ICI is strongly linked to the host's immune system and tumor immune microenvironment (TIME). The immunotherapeutic response to ICI therapy represented by CTLA-4/PD-1 inhibitors in the high and low m^6^Ascore groups was evaluated in this study. Patients with a high m^6^Ascore had higher ICI scores in the anti-CTLA-4 therapy cohort alone, according to the findings. In both the anti-PD-1 therapy alone and the anti-CTLA-4/PD-1 treatment cohorts, patients with a low m6Ascore had higher ICI scores. Therefore, the m^6^Ascore could be used in the future to evaluate the efficacy of the clinical responses of patients to immunotherapy.

However, our study has few limitations which are as follow: our conclusions are mainly based on bioinformatics methods, so further experiments and clinical verification are needed; There is a certain overlap between the m6Acluster subtypes that we have identified, so this method has certain limitations for some patients, and further development of more complete methods is needed in the future.

## Conclusion

In conclusion, this study elucidated several extensive regulatory mechanisms underlying m^6^A methylation modification in LUAD. An m^6^A-scoring signature was created to identify m^6^A modification patterns in individual tumors. The heterogeneity of m^6^A modification patterns was highlighted, and the findings may enhance the understanding of the characterization of the tumor microenvironment and guide the development of effective immunotherapeutic strategies in the future.

## Supplementary Information

Supplementary Information 1.

Supplementary Information 2.

Supplementary Information 3.

Supplementary Information 4.

Supplementary Information 5.

Supplementary Information 6.

## Data Availability

The data sets analysed during the current study are available in the TCGA (https://portal.gdc.cancer.gov/) and GEO repository (https://www.ncbi.nlm.nih.gov/geo/).

## Abbreviations

LUAD: Lung adenocarcinoma
m^6^A: N6-methyladenosine
TCGA: The Cancer Genome Atlas
GEO: Gene-Expression Omnibus
FPKM: Fragments per kilobase million
TPM: Transcripts per million
TCIA: The Cancer Immunome Database
ICI: Immune checkpoint inhibitor
TIME: Tumor immune microenvironment
