# Supplementary material for: Analysis of m6A modulator-mediated methylation modification patterns and the tumor microenvironment in lung adenocarcinoma
Source: Sci Rep. 2022 Nov 30;12:20684. doi: 10.1038/s41598-022-20730-6 (PMC9712433; doi:10.1038/s41598-022-20730-6)
Supplement: Supplementary file 1 — Supplementary Information 1. [file 41598_2022_20730_MOESM1_ESM.docx]

**Figure S1: Flow chart.**

**Figure S2: There was no significant difference in the expression of m^6^A regulators in the ZC3H13 wild group and the ZC3H13 mutation group.**

**Figure S3: Independent prognostic analysis.**

(A) Univariate Cox analysis. (B) Multivariate COX analysis.

**Figure S4: Correlation analysis of tumor microenvironment and clinic.**

The clinical correlation of ESTIMATEScore (A), StromalScore (B) and ImmuneScore (C).

**TABLE S1: Clinical Information.**

**TABLE S2: Clinical information of TCGA patients in the high- and low- m^6^Asocre group.**
