# Supplementary material for: Analysis of m6A modulator-mediated methylation modification patterns and the tumor microenvironment in lung adenocarcinoma
Source: Sci Rep. 2022 Nov 30;12:20684. doi: 10.1038/s41598-022-20730-6 (PMC9712433; doi:10.1038/s41598-022-20730-6)
Supplement: Supplementary file 2 — Supplementary Information 2. [file 41598_2022_20730_MOESM2_ESM.docx]

**TABLE S1: Clinical Information.**

| Covariates | Type | TCGA-LUAD | GSE68465 |
| --- | --- | --- | --- |
| Age | <=55 | 80 (15.6%) | 81 (18.3%) |
| Age | >55 | 423 (82.5%) | 362 (81.7%) |
| Age | unknow | 10 (19.%) | 0 (0%) |
| Gender | female | 274 (53.4%) | 220 (49.7%) |
| Gender | male | 239 (46.6%) | 223 (50.3%) |
| Stage | Stage I | 275 (53.6%) | unknow |
| Stage | Stage II | 122 (23.8%) | unknow |
| Stage | Stage III | 82 (15.9%) | unknow |
| Stage | Stage IV | 26 (5.07%) | unknow |
| Stage | unknow | 8 (1.6%) | unknow |
| T | T1 | 172 (33.5%) | unknow |
| T | T2 | 274 (53.4%) | unknow |
| T | T3 | 45 (8.8%) | unknow |
| T | T4 | 19 (3.7%) | unknow |
| T | unknow | 3 (0.58%) | unknow |
| M | M0 | 344 (67.1%) | unknow |
| M | M1 | 25 (4.8%) | unknow |
| M | unknow | 144 (28.1%) | unknow |
| N | N0 | 330 (64.3%) | unknow |
| N | N1 | 97 (18.9%) | unknow |
| N | N2 | 72 (14.0%) | unknow |
| N | N3 | 2 (0.39%) | unknow |
| N | unknow | 12 (2.3%) | unknow |

Unknow: Data missing/unavailable.

**TABLE S2: Clinical information of TCGA patients in the high- and low- m^6^Asocre group.**

| Covariates | Type | High- m^6^Asocre | Low- m^6^Asocre |
| --- | --- | --- | --- |
| Age | <=55 | 39 (17.4%) | 22 (19.5%) |
| Age | >55 | 185 (82.6%) | 91 (80.5) |
| Gender | female | 117 (52.2%) | 54 (47.8%) |
| Gender | male | 107 (47.8%) | 59 (52.2%) |
| Stage | Stage I-II | 179 (79.9%) | 76 (67.2%) |
| Stage | Stage III-IV | 45 (20.1%) | 37 (32.7%) |
| T | T1-2 | 195 (87.1%) | 95 (84.1%) |
| T | T3-4 | 29 (12.9%) | 18 (15.9%) |
| M | M0 | 210 (93.8) | 104 (92.0%) |
| M | M1 | 14 (6.2%) | 9 (8.0%%) |
| N | N0-1 | 197 (87.9%) | 86 (76.1%) |
| N | N2-3 | 27 (12.1%) | 27 (23.9%) |
