# Supplementary figures and images for: Analysis of m6A modulator-mediated methylation modification patterns and the tumor microenvironment in lung adenocarcinoma
Source: Sci Rep. 2022 Nov 30;12:20684. doi: 10.1038/s41598-022-20730-6 (PMC9712433; doi:10.1038/s41598-022-20730-6)

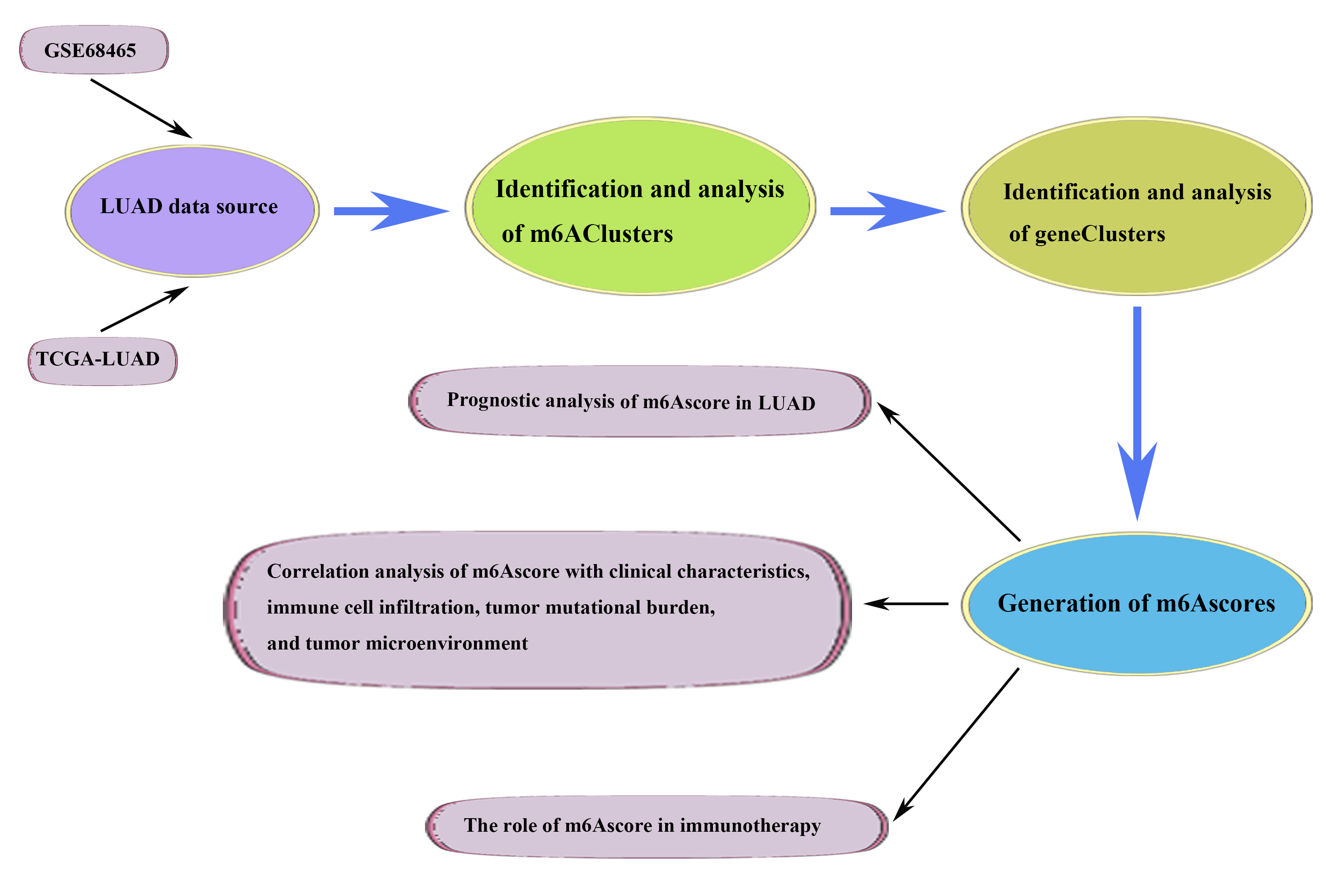

Supplement: Supplementary file 3 — Supplementary Information 3. [file 41598_2022_20730_MOESM3_ESM.tif]

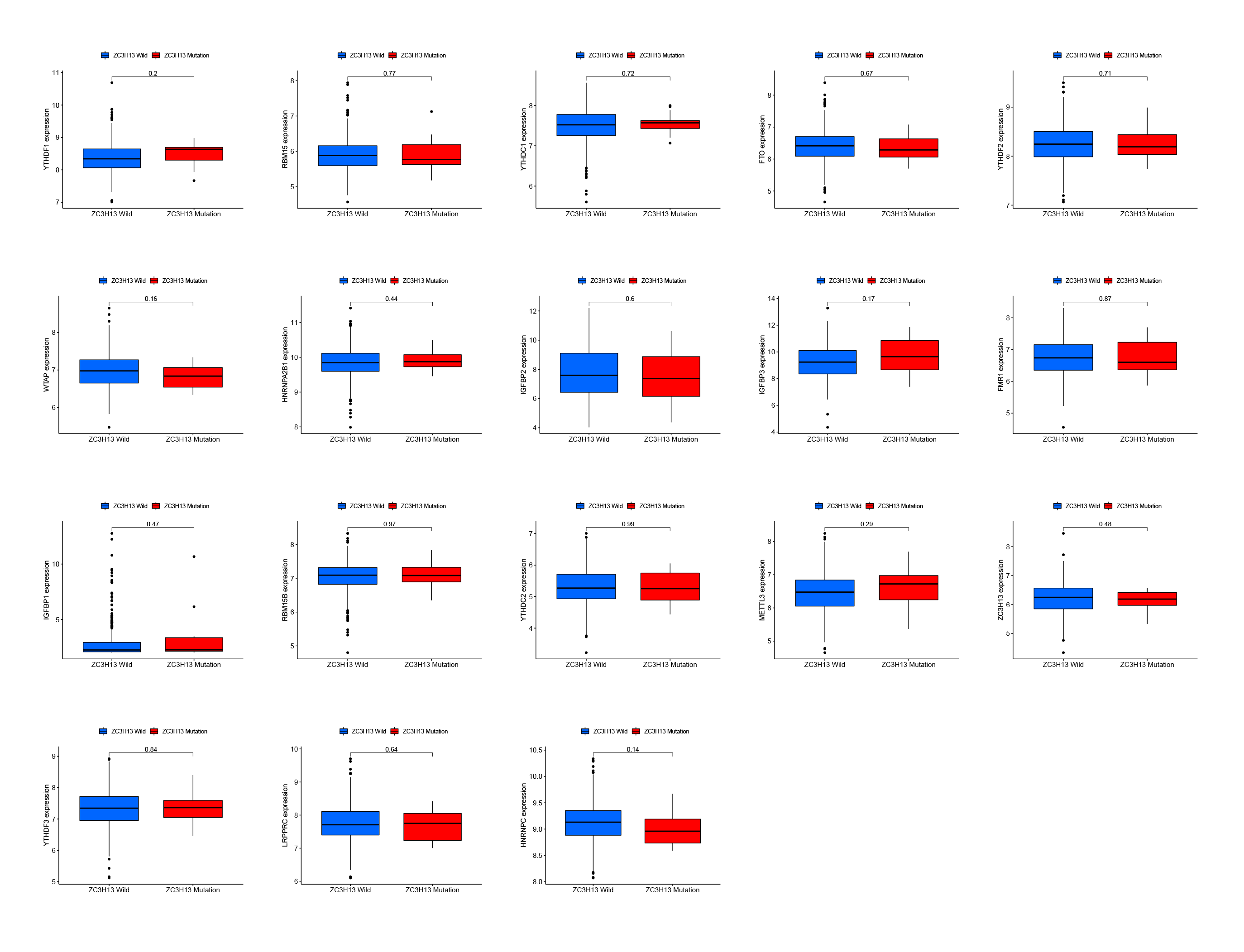

Supplement: Supplementary file 4 — Supplementary Information 4. [file 41598_2022_20730_MOESM4_ESM.tif]

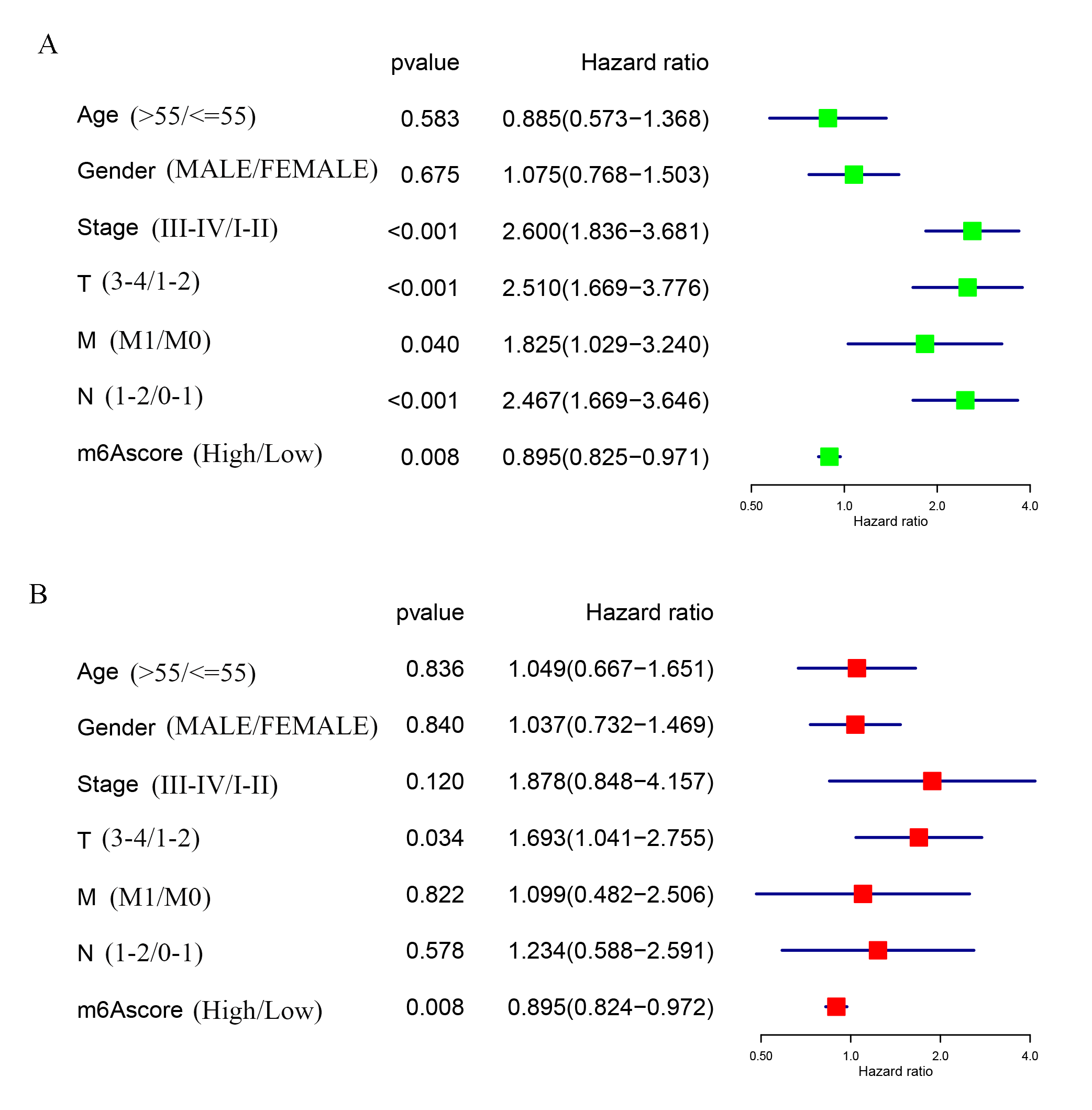

Supplement: Supplementary file 5 — Supplementary Information 5. [file 41598_2022_20730_MOESM5_ESM.tif]

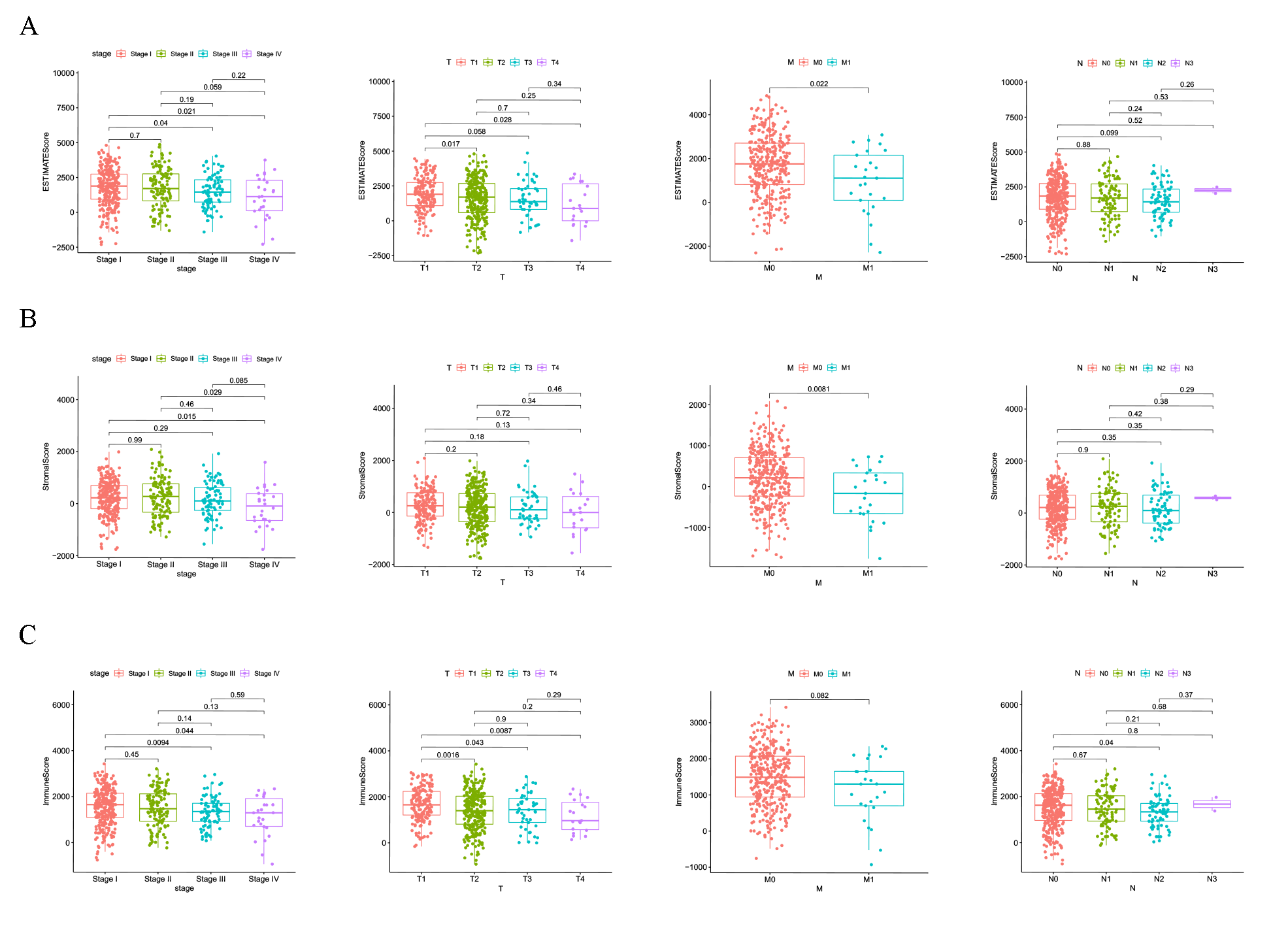

Supplement: Supplementary file 6 — Supplementary Information 6. [file 41598_2022_20730_MOESM6_ESM.tif]
